# Supplementary material for: Chromothripsis during telomere crisis is independent of NHEJ, and consistent with a replicative origin
Source: Genome Res. 2019 May;29(5):737–49. doi: 10.1101/gr.240705.118 (PMC6499312; doi:10.1101/gr.240705.118)
Supplement: Supplemental Material [file supp_gr.240705.118_Supplemental_file_1.zip › contigs/annotated_contigs/DB56/contig.2.DB56_length_278_mean_cov_3.74100719424.docx]

**DB56_length_278_mean_cov_3.74100719424**

```
AAGGCAGCAGGATATATATATATATATATATATATATATATA|CAAACAGA|AATACAAACAGAAAAGTATTAGGTAAGAATATATTAG
 >chr13:92238742-92238784 + E=6e-14 p=2e-03        >chr13:92228176-92228404 + E=2e-126
ATGTCAATAATACAGTAATTTTGGCAACATTTATTTAGCATGAAAACCGATAATGTCAGAATATGAAAGAAGTAGTAGGTAGAACTGTC

CTGAATATTGTCAGTCATAGCAGTGTAGGATGGAGATTACCAGAGGCTGAAGGATGGGAACATTGGGCAGACAGATGTTGGTCAAAAGG

TACAAAATTTCAG
```
